# Helical and kinase domain mutations of *PIK3CA*, and their association with hormone receptor expression in breast cancer

**DOI:** 10.3892/ol.2019.10565

**Published:** 2019-07-04

**Authors:** Chittibabu Vatte, Ali Mohammed Al Amri, Cyril Cyrus, Shahanas Chathoth, Ahmed Alsayyah, Arafat Ahmad, Mohammed Shakil Akhtar, Nada Fehaid Alrashidi, Nithya Jayaseeli, Hamed Al Wadani, Alhussain Al Zahrani, Amein Kadhem Al Ali

**Affiliations:** 1 Department of Biochemistry, College of Medicine, Imam Abdulrahman Bin Faisal University, Dammam 31441, Kingdom of Saudi Arabia; 2 Department of Genetic Research, Institute for Research and Medical Consultations, Imam Abdulrahman Bin Faisal University, Dammam 31441, Kingdom of Saudi Arabia; 3 Department of Internal Medicine, King Fahd Hospital of The University, Imam Abdulrahman Bin Faisal University, Al-Khobar 31952, Kingdom of Saudi Arabia; 4 Department of Pathology, King Fahd Hospital of The University, Imam Abdulrahman Bin Faisal University, Al-Khobar 31952, Kingdom of Saudi Arabia; 5 Department of Surgery, King Faisal University, Hofuf, Al-Ahsa 31982, Kingdom of Saudi Arabia; 6 College of Medicine, King Faisal University, Hofuf, Al-Ahsa 31982, Kingdom of Saudi Arabia; 7 Department of Clinical Laboratories Sciences, College of Applied Medical Sciences, King Saud University, Riyadh 11362, Kingdom of Saudi Arabia

**Keywords:** somatic mutation, hormone receptor, prognosis, target therapy, sequencing

## Abstract

Breast cancer is one of the major causes of female morbidity and mortality, accounting for ~25% of the total cancer cases in women. Phosphatidylinositol-4,5-bisphosphate 3-kinase catalytic α subunit (*PIK3CA*) mutations serve a major role in downstream signaling of receptor tyrosine kinases. The present study aimed to elucidate the frequency of exon 9 and 20 mutations of *PIK3CA* and their role in disease progression. A total of 118 tumor samples from confirmed breast cancer patients were collected from the histopathology laboratory at King Fahd Hospital of the University (Al-Khobar, Saudi Arabia). Sanger sequencing was performed on extracted DNA to identify the mutations on exons 9 and 20 of *PIK3CA*. The results were further validated by competitive allele-specific TaqMan polymerase chain reaction. Three mutations, namely E542K and E545K within exon 9, and H1047R within exon 20, were observed in 25 patients (21.2%). Among these, 18 patients carried the H1047R mutation of the kinase domain, while the remaining 7 patients carried mutations in the helical domain. *PIK3CA* mutations were associated with the estrogen receptor-positive/progesterone receptor-positive (ER^+^/PR^+^) group of tumors in contrast to the ER^−^/PR^−^ group (P=0.021). Furthermore, it was observed that the *PIK3CA* mutation was associated with a poor disease prognosis. Taken together, the current study emphasized the potential of *PIK3CA* mutations as an important biomarker for breast cancer classification and the possible use of *PIK3CA* inhibitor as targeted therapy for breast cancer.

## Introduction

Breast cancer is the most frequently diagnosed cancer in women, accounting for ~25% of the total number of cancer cases, and is one of the leading causes of female mortalities worldwide (1). Breast cancer is categorized based on clinicopathological features and molecular signatures. Estrogen receptor (ER), progesterone receptor (PR) and human epidermal growth factor receptor 2 (HER2) serve as key biomarkers in breast cancer, and their expression in tumor cells forms the basis for endocrine therapy, targeted therapy and disease prognosis.

Phosphatidylinositol-4,5-bisphosphate 3-kinase catalytic subunit α (*PIK3CA*), which is located on chromosome 3q26.32, encodes the catalytic subunit p110α of class IA phosphoinositide 3-kinase (PI3K). *PIK3CA* is one of the most commonly mutated oncogenes in several types of human cancer, including breast, colon and endometrial cancer (2). It serves a major role in downstream signaling of receptor tyrosine kinases (RTKs), and is therefore critical in the regulation of cell proliferation, growth, differentiation, migration and survival (2,3). In 2004, somatic mutations in the *PIK3CA* gene were reported for the first time in solid tumors (3). Although the role of *PIK3CA* mutations in diagnosis and disease progression has been studied extensively (4–15), there is no consensus on the use of *PIK3CA* as a biomarker.

A previous study by Samuels *et al* (3) reported that the frequency of *PIK3CA* mutations in breast cancer was 8%, while further studies revealed that approximately 21–34% of breast cancer cases presented *PIK3CA* mutations (2,16,17). It has been reported that *PIK3CA* somatic mutations are clustered within exons 9 and 20, which correspond to the helical and kinase domains, respectively (2,18,19), and are gain-of-function mutations with a transforming capacity (20). In addition, mutations in exon 9 (helical domain) are resistant to the inhibitory effect of p85 through Src-homology 2 domain. A higher level of mutations in the kinase domain (14.6%) compared with the helical domain was reported in a large cohort with predominantly lymph node-positive breast cancer (21).

Among Saudi women with cancer, the incidence of breast cancer ranges between 18 and 34.8%, with the lowest frequency observed in the southwestern region (18%) and the highest frequency in the eastern region (34.8%). The highest incidence of breast cancer was observed in women aged between 30–44 years (22). Therefore, the objective of the present study was to evaluate the *PIK3CA* hotspot mutations in breast cancer patients in Saudi Arabia and in subsets of breast cancer cases based on hormone receptor expression.

## Materials and methods

### 

#### Study population and histopathology

This retrospective study included a total of 118 breast tumor samples from 118 women who were histopathologically diagnosed with breast cancer (23) at King Fahd Hospital of the University (Al-Khobar, Saudi Arabia). The inclusion criteria were: i) Confirmed breast cancer diagnosis by histopathology analysis; and ii) patient age, 18–80 years. The tumor samples were collected between May 2005 and February 2014, and the mean age of the patients was 50.26±11.36 years. All patients were from the eastern province of Saudi Arabia. The formalin-fixed paraffin-embedded (FFPE) tissue specimens were subjected to hematoxylin and eosin staining to examine the tumor cell content and reconfirm the diagnosis. FFPE specimens containing >70% of tumor cells were selected for further analysis. The clinicopathological parameters and expression pattern of ER, PR and HER2 determined by immunohistochemistry were obtained from the medical records of the patients. Patient outcome was estimated according to the status of 12 parameters, including local recurrence, absence of ER and PR expression, grade 3 tumor, stage III and IV tumors, Ki67 expression (>50%), lymph node positivity, visceral metastasis, lympho-vascular invasion, age (<50 years), tumor size (>5 cm) and HER2 positivity. The prognosis of patients exhibiting ≥8 of the aforementioned characteristics was considered to be poor, while patients with <8 of these characteristics were considered to have good prognosis. Based on the ER, PR and HER2 expression pattern of the tumor tissues the patient cohort was divided into 26 groups, including the ER^+^, ER^−^, PR^+^, PR^−^, HER2^+^, HER2^−^, ER^−^PR^−^, ER^+^PR^+^, ER^−^PR^+^, ER^+^PR^−^, ER^−^HER2^−^, ER^+^HER2^+^, ER^−^HER2^+^, ER^+^HER2^−^, PR^−^HER2^−^, PR^+^HER2^+^, PR^−^HER2^+^, PR^+^HER2^−^, ER^−^PR^−^HER2^−^, ER^−^PR^+^HER2^−^, ER^+^PR^+^HER2^−^, ER^+^PR^+^HER2^+^, ER^+^PR^−^HER2^+^, ER^+^PR^−^HER2^−^, ER^−^PR^−^HER2^+^ and ER^−^PR^+^HER2^+^ groups.

The current study was approved by the Institutional Review Board of Imam Abdulrahman Bin Faisal University (approval no. IRB-2014-01-007; Dammam, Saudi Arabia). Written informed consent was obtained from patients for the use of FFPE specimens for research purposes.

#### Detection of PIK3CA mutations by direct sequencing

**Figure 1.**
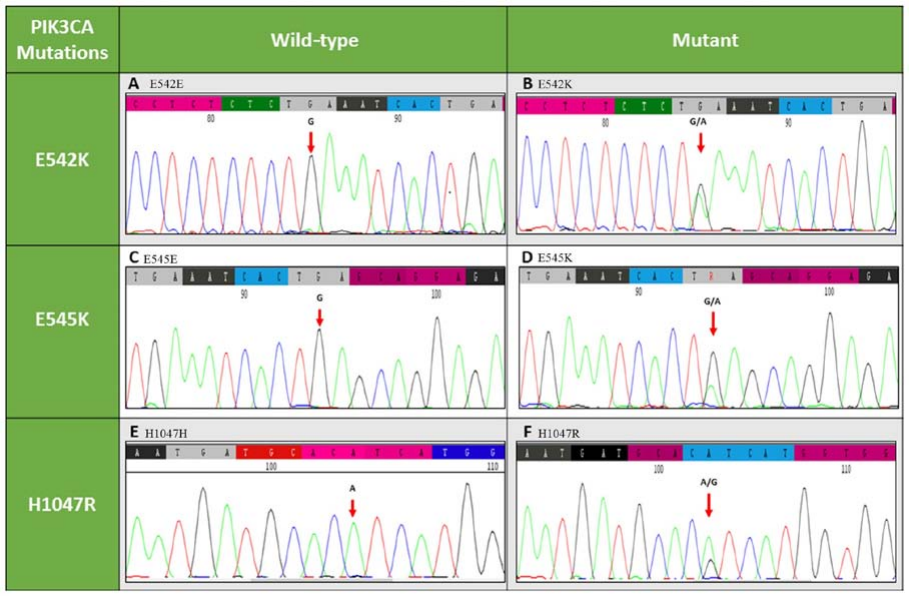
Representative electropherograms of exons 9 and 20 of *PIK3CA* gene. Graphs are shown for (A) E542E (wild-type), (B) E542K (mutant), (C) E545E (wild-type), (D) E545K (mutant), (E) H1047H (wild-type) and (F) H1047R (mutant). *PIK3CA*, phosphatidylinositol-4,5-bisphosphate 3-kinase catalytic α subunit.

The FFPE tissue specimens were subjected to microtomy to obtain 5-µm sections. Six sections from each specimen were utilized for DNA isolation using the QIAmp DNA FFPE tissue kit (Qiagen GmbH, Hilden, Germany) as per the manufacturer's protocol. The purified DNA was checked for DNA quantity and quality by Nanodrop spectrophotometer (Thermo Fisher Scientific, Inc., Waltham, MA, USA). Mutations in exons 9 and 20 of *PIK3CA* were detected by polymerase chain reaction (PCR) in a total volume of 25.0 µl, containing 1.25 U GoTaq polymerase (Promega Corporation, Madison, WI, USA), 0.2 mM of each dNTP, 1.5 mM MgCl_2_, 1X Taq buffer, 20 pM of each primer (exon 9 forward, 5′-CCAGAGGGGAAAAATATGACA-3′; exon 9 reverse, 5′-CATTTTAGCACTTACCTGTGAC-3′; exon 20 forward, 5′-CATTTGCTCCAAACTGACCA-3′; and exon 20 reverse, 5′-TGAGCTTTCATTTTCTCAGTTATCTTTTC-3′) and 50 ng DNA. The thermocycling conditions were as follows: 1 cycle of 5 min at 95°C; 40 cycles of 30 sec at 95°C, 30 sec at 54°C, and 30 sec at 72°C; and 1 cycle of 5 min at 72°C. Subsequently, the PCR products were analyzed on a 2% agarose gel, which indicated the expected amplicon for exon 9 of 195 bp and for exon 20 of 338 bp (24). The sequence analysis was performed using the CodonCode aligner software (CodonCode Corporation, Centerville, MA, USA). The NG_012113.2 sequence (National Center for Biotechnology Information, Bethesda, MD, USA) was used as a reference sequence. The helical and kinase mutations E542K, E545K and H1047R are represented in Fig. 1.

#### Detection of PIK3CA mutations by competitive allele-specific TaqMan (CAST) PCR

The mutation results were confirmed using CAST PCR technology with the ABI 7500 Real-time PCR thermocycler (Thermo Fisher Scientific, Inc.). A total of three TaqMan mutation detection assays (Thermo Fisher Scientific, Inc.) for E542K (assay ID, Hs00000822_mu; cat. no. 4465804), E545K (assay ID, Hs00000824_mu; cat. no. 4465804) and H1047R (assay ID, Hs00000831_mu; cat. no. 4465804) were performed using quantitative PCR as per the manufacturer's protocol. Briefly, 1X TaqMan genotyping master mix, locus specific TaqMan FAM dye labeled mutation assay and 50 ng DNA were mixed, made up to 20 µl final volume in a 96-well plate, and subjected to quantitative PCR on the ABI7500 Real-time PCR system using the following thermocycling conditions: 95°C for 10 min, followed by five cycles at 92°C for 15 sec and 58°C for 1 min, and forty cycles at 92°C for 15 sec and 60°C for 1 min. Simultaneously, reference *PIK3CA* was amplified using a reference assay probe. Mutation Detector™ software (version 2.0; Thermo Fisher Scientific, Inc.) was used to classify the mutation status. The tumor samples that did not exhibit these three mutations were used as negative control samples in CAST PCR.

#### Statistical analysis

The frequency of each mutation was confirmed by direct counting. Fisher's exact test was used to determine the significance of the association among the groups. A statistically significant difference was determined if the P-value was <0.05. SPSS version 19 (IBM Corp., Armonk, NY, USA) software was used to perform the Fisher's exact test. Survival analysis was performed using Kaplan-Meier curve analysis and log-rank test.

## Results

### 

#### Clinical and histopathological characteristics

The mean age of the patient population was 50.26±11.36 years. Histopathological analysis revealed that the majority of the patients presented with grade 2 and stage 2 breast cancer, and the patient prognosis was ascertained based on 12 parameters, as described earlier (≥8 parameters indicated poor, and <8 parameters indicated good prognosis; Table I). Ductal carcinoma was observed in 95.76% of the patients, whereas only 4.2% of the patients presented with lobular carcinoma. Based on ER, PR and HER2 expression levels, it was determined that 62.7% of the cases exhibited high ER expression and 50% exhibited low PR expression, while 74.6% of patients were HER2^−^. In the patient cohort, 45% of cases were ER^+^/PR^+^, while 64.4% were ER^−^/PR^−^. The lowest frequency of two marker positive expression was seen in the PR^+^/HER2^+^ and ER^+^/HER2^+^ groups at 7.6 and 9.3% respectively. When the patients were classified into the different marker groups based on ER, PR and HER2 expression, the majority of the cases were assigned to the ER^+^/PR^+^ and HER2^−^ group (39%), with the ER^−^/PR^+^ and HER2^+^ group exhibiting the lowest frequency (1.7%).

#### PIK3CA helical and kinase domain mutations

Three mutations, namely E542K and E545K within exon 9, and H1047R within exon 20, were observed in 25 of the 118 patients (21.2%; Table I). Among these 25 patients, 3 carried the E542K helical domain mutation, while 4 carried the E545K helical domain mutation (data not shown). The remaining 18 patients carried the H1047R mutation of the kinase domain (Table I). This indicated that the frequency of the kinase domain mutation was significantly greater when compared with the helical domain mutation (P=0.019; data not shown). The patients were then classified into two groups based on good prognosis (53%) or poor prognosis (46.6%). Accordingly, it was observed that the *PIK3CA* mutation was significantly associated with poor prognosis (P=0.023). Furthermore, once the patient cohort was stratified based on the expression of the three receptors (ER, PR and HER2), *PIK3CA* mutations were associated with the ER^+^/PR^+^ group of tumors in contrast to the ER^−^/PR^−^ group (P=0.021).

The helical and kinase domain mutations were independently analyzed to determine the association with histopathological and prognostic indicators. This analysis revealed that there was no independent association of the indicators, whereas the *PIK3CA* mutation was overall associated with a poor prognosis. A significant association was identified between the ER^+^/PR^+^ subgroup and the H1047R kinase domain mutation of *PIK3CA* (P=0.038); however, no association with the helical domain mutations was identified (Table II).

## Discussion

RTK signaling induces the activation of Ras/mitogen-activated protein kinase and the Ras/PI3K/protein kinase B (AKT) signaling pathways, resulting in increased proliferation, survival and metastasis of cancer cells. Somatic mutations in RTKs, Ras, B-Raf, PI3K and AKT are commonly observed in various cancer types (25,26). Thus, research has focused on the genetic alterations in the genes involved in these pathways for use in targeted therapy. Samuels *et al* (3) identified *PIK3CA* gene mutations in a small study cohort and suggested that these mutations may be involved in the development of cancer. These mutations were primarily concentrated in the helical, kinase and p85 binding domains. Subsequent studies in different population groups revealed that the frequency of these mutations ranged between 21.3 and 34.7% (2,16). A study by Karakas *et al* (27) conducted on a central Saudi Arabian population reported the frequency of these mutations to be 26%. The present study indicated a frequency of 21.2% for these mutations in breast cancer patients in the eastern province of Saudi Arabia, which was comparable with the findings of Karakas *et al* (27). Furthermore, the current study revealed that the frequency of mutations in exons 9 and 20 was 5.9 and 15.25%, respectively. However, none of the patients included in the current study presented with these two mutations simultaneously. These results are in line with previously reported studies in other population groups (2,16,28,29).

According to the current study data, it can be suggested that *PIK3CA* mutations are observed in similar percentages worldwide. However, the higher frequency of kinase domain mutations in the present study, particularly H1047R, suggested the need for targeted therapy to inhibit *PIK3CA* activity via the H1047R mutation. A number of studies have investigated the association between these mutations and clinicopathological parameters, including hormone receptor expression, stage, grade, metastases and prognosis of breast cancer (4–8). A previous meta-analysis comprising 32 different studies reported that ER and PR expression levels were significantly correlated with *PIK3CA* mutations (P<0.00001) (4). HER2-overexpressing breast tumors were correlated with a high *PIK3CA* mutation rate (5). A study by Li *et al* (6) confirmed that there was a correlation between *PIK3CA* mutations, and the ER and PR overexpression in large tumors. The present study results were in line with these aforementioned studies, and reinforced the correlation of ER and PR overexpression with the high frequency of *PIK3CA* mutations (P=0.021). However, ER and PR overexpression were significantly correlated with the kinase domain mutation (H1047R; P=0.038) rather than the helical domain mutations (P=0.401). It has also been reported that ER^+^ tumors were associated with a higher frequency of *PIK3CA* mutations and that ER could be activated in the absence of estrogen, which causes tamoxifen resistance (7). Furthermore, a study by Ellis *et al* (8) demonstrated that tumors with *PIK3CA* mutations did not respond to neoadjuvant endocrine therapy as compared with tumors without *PIK3CA* mutations.

**Figure 2.**
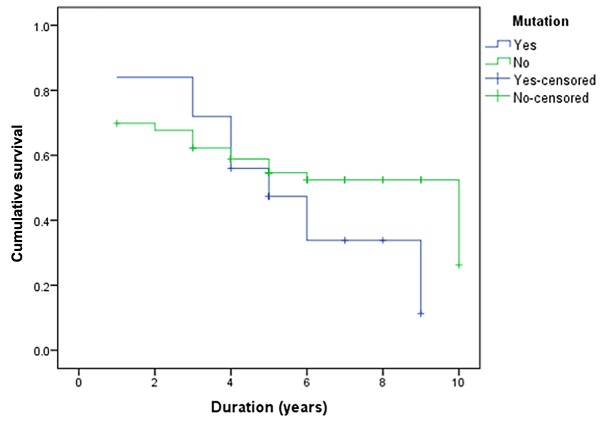
Kaplan-Meier curve comparing the survival of the patients with and without *PIK3CA* mutation. Censored refers to cases with incomplete survival data or loss to follow up. *PIK3CA*, phosphatidylinositol-4,5-bisphosphate 3-kinase catalytic α subunit.

Several retrospective and prospective studies have evaluated the prognostic and predictive value of *PIK3CA* mutations in breast cancer tumors. However, these studies reached contradictory conclusions, with certain studies demonstrating the association of *PIK3CA* mutations with poor survival (9,10), while others indicating that *PIK3CA* mutations was associated with a better survival rate (11,12). In addition, a study by Barbareschi *et al* (13) reported that only exon 9 mutations were associated with poor survival. Furthermore, a meta-analysis comprising seven studies revealed that *PIK3CA* mutations have no prognostic impact (4). In this previous meta-analysis, the prognosis was defined by overall survival and progression-free survival. Similarly, in the present study, the *PIK3CA* mutations did not yield a significant impact on overall survival (P=0.248; Fig. 2). However, sub-data analysis based on 12 parameters indicated that the *PIK3CA* mutations within exons 9 and 20 were associated with poor prognosis (P=0.023). Several studies revealed variation in the significant correlation between *PIK3CA* mutation and overall survival (9–13), while the data of the present study indicated no significant impact. The possible reasons may include the different exonic mutations that may impact different mechanisms, the breast cancer-specific effect of *PIK3CA* mutations (4), and the impact of treatment, which greatly varies subsequent to recurrence (17).

Recent clinical and experimental studies suggested that PI3K pathway activation may negatively affect the response of breast cancer patients to trastuzumab, a monoclonal therapy drug (2). This conclusion emphasized the need to assess the *PIK3CA* mutation status following trastuzumab therapy for breast cancer in order to predict disease progression (14,15). Currently, only one drug that targets the PI3K pathway is available for breast cancer treatment, namely everolimus, which is an mTOR inhibitor. *PIK3CA* mutations may provide an opportunity to develop novel drugs that target altered *PIK3CA*, or combination therapy based on the current regimen, which may yield the maximum effect on breast tumors. Therefore, determining the *PIK3CA* mutation status has valuable clinical relevance in terms of disease prediction and prognosis.

Different detection methods exist to identify mutations in a gene. The standard method is direct sequencing, which identifies existing and *de novo* mutations, unlike amplification-refractory mutation system PCR or quantitative PCR-based methods (30). All these methods have their respective sensitivity in detecting the mutation status. The vast majority of studies have employed Sanger sequencing to determine *PIK3CA* mutations (4). Based on the meta-analysis conducted by Pang *et al* (4) on *PIK3CA* mutations in breast cancer, 18 out of 26 studies determined the *PIK3CA* sequence by Sanger sequencing. Another study by Papaxoinis *et al* (21) reported a good concordance between direct sequencing and next-generation sequencing methods. The present study employed direct sequencing and a TaqMan mutation detection assay by CAST PCR. Initially, all the samples were subjected to direct sequencing, followed by further validation of samples with *PIK3CA* mutations using CAST PCR. All *PIK3CA* mutation positive samples and 10% of the *PIK3CA* mutation negative samples assessed using CAST PCR recorded 100% concordance with direct sequencing results. Thus, both Sanger sequencing and CAST PCR assays can be used to detect *PIK3CA* mutations. However, CAST PCR may be a preferred method due to high detection sensitivity, low cost and time efficiency.

In conclusion, the present study emphasized that *PIK3CA* mutations may serve as important biomarkers for breast cancer classification and for targeted therapies using PIK3CA inhibitors.

## Figures and Tables

**Table I.** Association of baseline characteristics with the *PIK3CA* helical and kinase mutation status.

		All cases, n			*PIK3CA* helical, n			*PIK3CA* kinase, n		
				
Category	Subjects, n (%)	*PIK3CA* mutation	No *PIK3CA* mutation	P-value	Mutation	No mutation	P-value	Mutation	No mutation	P-value
Age, years										
≤50	66 (55.9)	15	51	0.820	4	62	>0.999	11	55	0.797
>50	52 (44.1)	10	42		3	49		7	45	
Histological grade										
Grade 1	14 (11.86)	1	13		0	14		1	13	
Grade 2	69 (58.47)	15	54	0.285	4	65	>0.999	11	58	0.680
Grade 3	35 (29.66)	9	26	0.243	3	32	0.547	6	29	0.656
Histological stage										
Stage 1	14 (11.86)	2	12		1	13		1	13	
Stage 2	58 (49.15)	12	46	0.722	2	56	0.482	10	48	0.679
Stage 3	34 (28.81)	9	25	0.469	3	31	>0.999	6	28	0.656
Stage 4	12 (10.17)	2	10	>0.999	1	11	>0.999	1	11	1
Prognosis										
Good	63 (53.39)	8	55	0.023	2	61	0.248	6	57	0.076
Poor	55 (46.61)	17	38		5	50		12	43	

*PIK3CA*, phosphatidylinositol-4,5-bisphosphate 3-kinase catalytic α subunit.

**Table II.** Hormone receptor subsets, and the *PIK3CA* helical and kinase mutation status.

	All cases, n			*PIK3CA* helical, n			*PIK3CA* kinase, n		
			
Category	*PIK3CA* mutation	No *PIK3CA* mutation	P-value	Mutation	No mutation	P-value	Mutation	No mutation	P-value
ER^+^	17	57		4	70		13	61	
ER^−^	8	36	0.644	3	41	0.711	5	39	0.435
PR^+^	14	45		4	55		10	49	
PR^−^	11	48	0.652	2	57	0.679	8	51	0.608
HER2^+^	6	24		3	27		3	27	
HER2^−^	19	69	>0.999	4	84	0.368	15	73	0.556
ER^−^PR^−^	6	70		2	74		4	72	
ER^+^PR^+^	12	41	0.021	3	50	0.401	9	44	0.038
ER^−^PR^+^	6	32	0.210	2	36	0.599	4	34	0.437
ER^+^PR^−^	5	16	0.056	1	20	0.523	4	17	0.064
ER^−^HER2^−^	5	20		1	24		4	21	
ER^+^HER2^+^	3	8	0.678	1	10	0.523	2	9	>0.999
ER^−^HER2^+^	3	16	>0.999	2	17	0.569	1	18	0.370
ER^+^HER2^−^	14	49	>0.999	3	60	>0.999	11	52	>0.999
PR^−^HER2^−^	7	31		2	36		5	33	
PR^+^HER2^+^	2	7	>0.999	2	7	0.160	0	9	0.566
PR^−^HER2^+^	4	17	>0.999	1	20	>0.999	4	17	0.707
PR^+^HER^2^−	12	38	0.607	2	48	>0.999	10	40	0.568
ER^−^PR^−^HER2^−^	4	17		1	20		3	18	
ER^−^PR^+^HER2^−^	1	3	>0.999	0	4	>0.999	1	3	0.526
ER^+^PR^+^HER2^−^	11	35	0.760	2	44	>0.999	9	37	0.739
ER^+^PR^+^HER2^+^	1	6	>0.999	1	6	0.444	0	7	0.551
ER^+^PR^−^HER2^+^	2	2	0.234	0	4	>0.999	2	2	0.166
ER^+^PR^−^HER2^−^	3	14	>0.999	1	16	>0.999	2	15	>0.999
ER^−^PR^−^HER2^+^	2	15	0.672	1	16	>0.999	1	16	0.613
ER^−^PR^+^HER2^+^	1	1	0.395	1	1	0.170	0	2	>0.999

Fisher's exact test was used to calculate the statistical significance. *PIK3CA*, phosphatidylinositol-4,5-bisphosphate 3-kinase catalytic α subunit; ER, estrogen receptor; PR, progesterone receptor; HER2, human epidermal growth factor receptor 2.

## Data Availability

The datasets used and/or analyzed during the current study are available from the corresponding author on reasonable request.
